# Soluble PCSK9 Inhibition: Indications, Clinical Impact, New Molecular Insights and Practical Approach—Where Do We Stand?

**DOI:** 10.3390/jcm12082922

**Published:** 2023-04-18

**Authors:** Michele Bellino, Gennaro Galasso, Angelo Silverio, Michele Tedeschi, Ciro Formisano, Stefano Romei, Luca Esposito, Francesco Paolo Cancro, Maria Giovanna Vassallo, Giulio Accarino, Monica Verdoia, Francesca Maria Di Muro, Carmine Vecchione, Giuseppe De Luca

**Affiliations:** 1 Department of Medicine, Surgery and Dentistry, University of Salerno, 84081 Baronissi, Italy; 2 Division of Cardiology, Ospedale Degli Infermi, ASL Biella, 13900 Biella, Italy; 3 Structural Interventional Cardiology, Department of Clinical and Experimental Medicine, Clinica Medica, Careggi University Hospital, 50139 Florence, Italy; 4 Vascular Physiopathology Unit, IRCCS Neuromed Mediterranean Neurological Institute, 86077 Pozzilli, Italy; 5 Division of Cardiology, AOU “Policlinico G. Martino”, Department of Clinical and Experimental Medicine, University of Messina, 98166 Messina, Italy; 6 Division of Cardiology, IRCCS Hospital Galeazzi-Sant’Ambrogio, 20161 Milan, Italy

**Keywords:** cardiovascular prevention, CAD, lipidology, LDL, PCSK9, precision medicine

## Abstract

Current research on cardiovascular prevention predominantly focuses on risk-stratification and management of patients with coronary artery disease (CAD) to optimize their prognosis. Several basic, translational and clinical research efforts aim to determine the etiological mechanisms underlying CAD pathogenesis and to identify lifestyle-dependent metabolic risk factors or genetic and epigenetic parameters responsible for CAD occurrence and/or progression. A log-linear association between the absolute exposure of LDL cholesterol (LDL-C) and the risk of atherosclerotic cardio-vascular disease (ASCVD) was well documented over the year. LDL-C was identified as the principal enemy to fight against, and soluble proprotein convertase subtilisin kexin type 9 (PCSK9) was attributed the role of a powerful regulator of blood LDL-C levels. The two currently available antibodies (alirocumab and evolocumab) against PCSK9 are fully human engineered IgG that bind to soluble PCSK9 and avoid its interaction with the LDLR. As documented by modern and dedicated “game-changer” trials, antibodies against soluble PCSK9 reduce LDL-C levels by at least 60 percent when used alone and up to 85 percent when used in combination with high-intensity statins and/or other hypolipidemic therapies, including ezetimibe. Their clinical indications are well established, but new areas of use are advocated. Several clues suggest that regulation of PCSK9 represents a cornerstone of cardiovascular prevention, partly because of some pleiotropic effects attributed to these newly developed drugs. New mechanisms of PCSK9 regulation are being explored, and further efforts need to be put in place to reach patients with these new therapies. The aim of this manuscript is to perform a narrative review of the literature on soluble PCSK9 inhibitor drugs, with a focus on their indications and clinical impact.

## 1. Avoidable Cardiovascular Risk: Global Burden, Target of Therapy and New Frontiers

In the context of a highly dynamic scenario with substantial medical achievements, coronary artery disease (CAD) remains the leading cause of mortality worldwide [1,2]. Therefore, current research predominantly focuses on the efficient prevention, risk-stratification, and management of patients with CAD to optimize their prognosis. Concurrently, several basic, translational and clinical research efforts aim to determine the etiological mechanisms underlying CAD pathogenesis [3,4,5,6,7,8,9,10] and to identify lifestyle-dependent metabolic risk factors or genetic and epigenetic parameters responsible for CAD occurrence and/or progression [11]. Thereby, clinicians could ultimately develop feasible and accurate risk assessment and prediction models with the potential to be incorporated into routine clinical practice.

Low-density lipoprotein (LDL) is the most widely recognized and studied modifiable risk factor associated with atherosclerotic cardiovascular disease (ASCVD). A log-linear association between the absolute exposure of LDL cholesterol (LDL-C) and the risk of ASCVD has been well documented over the years due to prospective cohort studies and randomize clinical trials [12,13,14,15,16].

LDL particle concentration can be lowered by reductions in saturated fat consumption and caloric intake, as well as by multiple classes of cholesterol-lowering therapies that have gone through continuous updates and improvements [17,18].

Efforts made to reduce LDL-C levels, manage major cardiovascular risk factors and identify new primary and secondary prevention targets aim to stabilize coronary plaques and consequently reduce cardiovascular events. Unstable coronary plaques that are prone to rupture and cause a wide range of immediate and long-term adverse cardiac events are characterized by large plaque burden, large lipid content, and thin fibrous caps. It is well established that statins can slow the progression of coronary atherosclerosis, reducing the inflammatory plaque burden. However, the effect of novel therapeutic targets, such as lipoprotein (a) (Lp(a)) and proprotein convertase subtilisin kexin type 9 (PCSK9), on plaque burden, composition and inflammatory pathways involved remains largely unknown [19,20].

PCSK9 was implicated in cholesterol metabolism, as shown by converging lines of investigation from the early 2000s. Cohen et al.’s experiments, performed in 2005, revealed that PCSK9 loss of function (LOF) gene variants were associated with lower LDL-C levels [21]. Individuals with PCSK9 LOF variants, who benefit from a lifetime of low LDL-C levels, also had reduced ASCVD risk. Circulating levels of PCSK9 represent the strongest regulator of cholesterol trafficking in the body.

PCSK9, a proteolytic enzyme, indirectly regulates serum LDL-C by causing the destruction of LDL receptors. Physiologically, the complex PCSK9/LDL receptor (LDLR) is internalized and destroyed by lysosomes. A lower number of LDL-R molecules on the cell surface is associated with a higher level of plasma LDL-C.

Anti-PCSK-9 monoclonal antibodies prevent the binding of extracellular PCSK9 to LDL-R and prevent its internalization and degradation. The effect is the reduction of plasma LDL-C [22].

Although entry of PCSK9 into cells is dependent on the LDLR, the bulk of internalized PCSK9 remains intact inside the cells for several hours. This time lag suggests that the embrace between PCSK9 and LDLR does not lead to immediate targeting of both ligands and receptors to the lysosome and that perhaps a recycling circuit is present that allows a single molecule of PCSK9 to return to the surface and act on new LDLR molecules that recycle [23,24]. PCSK9 action during time, as its inhibition, justifies a powerful effect on circulating LDL particles.

Given that PCSK9 inhibition has changed the therapeutic approach to LDL-C reduction and the outcome of patients at cardiovascular risk, moreover with fast-changing therapeutic platforms, the aim of this manuscript is to perform a narrative review of the literature on soluble PCSK9 inhibitor drugs with a focus on their indications and clinical impact.

## 2. PCSK9 Inhibitors’ Pleiotropic Effects

Outcome benefits with PCSK9 inhibition are related mainly to LDL lowering. However, cardiovascular risk reduction appears to exceed the benefit exclusively derived from LDL-C reduction. Various hypotheses ascribe to PCSK9 inhibition effects beyond reductions in LDL-C level [25]. PCSK9 is expressed by various cell types that are naturally involved in inflammation, atherosclerosis, thrombosis, and oncogenesis; thus, the inhibition of this protease may have different pleiotropic effects.

(a) Anti-atherosclerotic effect: PCSK9 inhibitors reduce the concentrations of pro-inflammatory cytokines and increase the levels of interleukin-10 (an anti-inflammatory interleukin), decreasing the expression of TNF-α and the C-C chemokine receptor type 2 (CCR2) and thereby inhibiting the mechanisms that promote atherosclerosis. [26]. Moreover, PCSK9 monoclonal antibodies reduce the expression of NADPH oxidase, involved in oxidative stress [27], and lower the expression of membrane-adhesive molecules (ICAM and VCAM) interfering with the TLR4/NF-kB cascade [28].
(b) Stabilization of atherosclerotic plaque: PCSK9 inhibition reduces the necrotic core of atheroma, constituting one of the main players in plaque instabilization, induces autophagy of inflammatory cells, and favors elimination of necrotic cells. These mechanisms promote plaque stabilization [29]. However, it was also reported that PCSK9 has direct proinflammatory effects on vessels, possibly through effects on another member of the LDLR family, the LDLR-related protein 1, or LRP1, which exerts a strong regulatory effect on the inflammatory stance of plaque macrophages. Loss of LRP1 induces inflammation, and PCSK9 in the plaque reduces LRP1 levels [30,31]. Thus, therapeutic blockades of PCSK9 via monoclonal antibodies could have on coronary plaques a combination of positive and negative effects. The balance of these is difficult to assess in exclusively clinical studies, given the tremendously beneficial effect of plasma LDL-C reduction [32,33].
(c) Anti-aggregation and anticoagulant effects: PCSK-9 is involved in platelet activation and aggregation through indirect and direct stimulation of CD36 (scavenger receptor) and low-density lipoprotein receptor-1 (LOX-1) on the surface of platelets. CD36 is activated directly by PCSK-9, but also by LDL and ox-LDL, while LOX-1 is activated by inflammatory stimuli, and its expression is increased by PCSK-9. Platelet aggregation is also favored by stimulation of TLR2 on the cell surface through lipid–peroxide-modified phospholipids transported by Lp(a). Inhibition of PCSK-9, therefore, has an antithrombotic effect for several reasons: reducing levels of LDL and ox-LDL, reducing Lp(a), and decreasing LOX-1 expression [34]. PCSK-9 is involved in coagulation through the downregulation of the low-density lipoprotein receptor-related protein (LRP-1). This one physiologically decreases the levels of tissue factor and factor VIII [35].
(d) Antineoplastic effect: LDL-C and triglycerides have a negative impact on the risk of developing cancer. Mutations causing a decrease in PCSK-9 activity are associated with inhibition of the progression of colorectal and breast cancer, mainly, but not only, through a decrease in LDL-C concentration [36].
(e) PCSK9-I and sepsis: the Toll-like receptor (TLR) plays a key role in the immune response and is activated by lipid molecules associated with the pathogen cell walls (lipopolysaccharides, lipoteichoic acid and phospholipomannan). PCSK9 reduces the elimination of lipids by downregulating LDL-R, thereby promoting the development of sepsis and septic shock. Use of PCSK-9 inhibitors may reverse this pathway [37].

## 3. PCSK9 Monoclonal Antibodies

The two currently available antibodies (alirocumab and evolocumab) are fully human-engineered IgG antibodies that bind to soluble PCSK9 and avoid its interaction with the LDLR. A PCSK9-deficiency condition results in tremendous accumulation of LDLR on the membrane of hepatocytes and a vigorous clearance of LDL particles, which result in significant lowering of plasma LDL-C levels. The subcutaneous injection of alirocumab or evolocumab introduces antibodies that, within few hours, can capture not only all the circulating PCSK9 but also the newly secreted PCSK9 with a long-lasting effect, determining a tremendous reduction of LDL-C circulating levels. Alirocumab and evolocumab were initially approved by the United States Food and Drug Administration (FDA) “as an adjunct to diet and maximally tolerated statin therapy for treatment of adults with heterozygous familial hypercholesterolemia (HeFH) or clinical ASCVD who require additional lowering of LDL-C”, but for the scientific rewards conquered, the therapeutic indications of these two molecules have greatly expanded.

### Pharmacokinetics and Drug Interactions

Alirocumab is commonly administered at a dosage of 75 or 150 mg every two weeks and reaches maximum plasma concentration in a time range of 3 to 7 days, with a predominant distribution in the bloodstream and a bioavailability of about 85 percent [38].

For several months to date, a 300 mg formulation has also been available in some European countries in monthly administration, and its diffusion is progressively growing.

The plasma half-life of alirocumab is about 17–20 days. The interval is reduced to 12 days for patients on statin therapy due to the normal increase in PCSK9 expression induced by statin. It is assumed that alirocumab, after binding to PCSK9, is degraded to amino acids by the endothelial reticulum system. There is no need for dosage adjustment according to age, sex, weight, renal or mild-to-moderate hepatic failure. There are limited data for patients with severe hepatic or renal impairment [39].

On the other hand, evolocumab is administered at a dosage of 140 or 420 mg. At the 140 mg dosage, it reaches peak median serum concentrations in 3–4 days with an absolute bioavailability of 72%. A proportional increase in bioavailability was observed for higher dosages. Evolocumab has a prevalent distribution in the bloodstream and a half-life of 11–17 days. It is eliminated in two different phases. At low concentrations, elimination occurs via saturation of PCSK9 binding; at higher concentrations, it occurs via a nonsaturable proteolytic pathway. There is no need for dosage adjustment according to age, sex, weight, renal or mild-to-moderate hepatic insufficiency [40,41].

As for alirocumab, because of increased PCSK9 expression, increased binding to PCSK9 was observed for Evolocumab in patients on statin therapy [42].

## 4. PCSK9 Monoclonal Antibodies’ Indications for Cardiovascular Primary Prevention

PCSK9 monoclonal antibodies, as documented by modern and dedicated “game-changer” trials (Table 1), reduce LDL-C levels by at least 60 percent when used alone and up to 85 percent when used in combination with high-intensity statins and/or other hypolipidemic therapies, including ezetimibe. Specifically, alirocumab and evolocumab reduce LDL-C levels by 46–73% when compared with placebo and by 30% when compared with ezetimibe [43,44,45].

Both alirocumab and evolocumab showed great efficacy in reducing LDL-C levels in patients with high or very high cardiovascular risk factors, including those with diabetes mellitus and chronic kidney failure, reducing ASCVD events [21,23]. In addition, PCSK9i increases HDL-C levels and reduces triglyceride and Lp(a) levels.

In the context of primary cardiovascular prevention, great attention should be paid to familial heterozygous hypercholesterolemia (HeFH). HeFH is a monogenic codominant disease responsible for early onset CVD (55 years for males, 60 years for females) due to persistently extreme plasma LDL-C values throughout life. It is generally caused by LOF mutations affecting the LDLR or apoB genes, or by gain-of-function mutations in PCSK9 genes. About 95% of HeFH forms can be attributed to LDLR mutations. Hypolipidemic therapy in these patients should be initiated as soon as possible, considering that they have a very high cardiovascular risk profile.

According to the latest ESC 2021 guidelines for the prevention of cardiovascular risk, the use, in primary prevention, of PCSK9 monoclonal antibodies in patients younger than 70 years of age is indicated, with an evidence class IC, in patients with high-risk HeFH who do not reach the LDL-C target (LDL-C ≤ 70 mg/dL or ≤1.8 mmol/L) and who are already on the maximally tolerated statin dose and ezetimibe therapy. However, not all patients tolerate statins. In this case, PCSK9 monoclonal antibodies are recommended in addition to ezetimibe with evidence class IIb (34–35).

**Figure 1:**
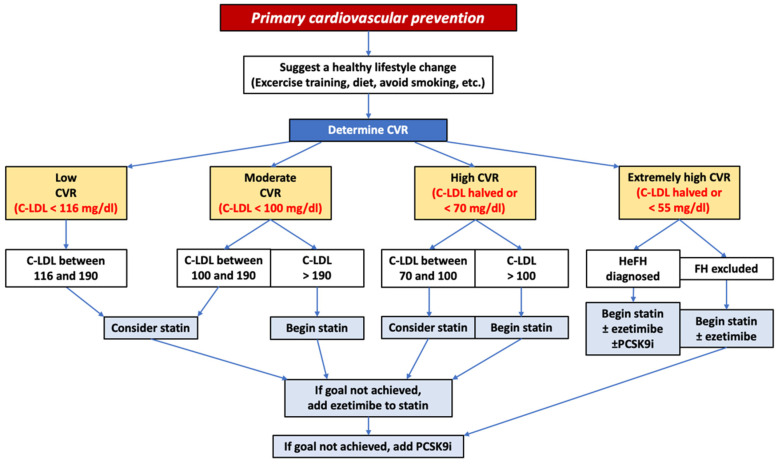
Flowchart of interventions for primary cardiovascular prevention according to the European Society of Cardiology guidelines. CVR: cardiovascular risk, C-LDL: LDL cholesterol, FH: familiar hypercholesterolemia.

Similarly, in patients without HeFH but with very high cardiovascular risk, already on maximally tolerated statin therapy and ezetimibe, a combination with PCSK9i (evidence class IIb) may be considered if the LDL-C target is not reached (Figure 1). In addition, 2018 AHA/ACC guidelines support the use of PCSK9i for primary prevention.

According to the reports, the use of PCSK9i should be evaluated in patients with HeFH aged 30 to 75 years, with LDL-C values of 100 mg/dL or higher (≥2.6 mmol/L), already on maximally tolerated statin dose and ezetimibe, and not reaching the desired therapeutic target [69,70].

Similarly, the use of PCSK9i can be evaluated in patients aged 40 to 75 with LDL-C at baseline of 200 mg/dL or higher (≥5.7 mmol/L), already on maximally tolerated statin therapy and ezetimibe, with achieved LDL-C values of 130 mg/dL or higher (≥3.4 mmol/L) [71].

On the other hand, homozygous FH (HoFH) is a rare genetic syndrome presenting with complete absence of functioning LDL-R. In this case, use of conventional therapy (statin, ezetimibe, PCSK9-i), which acts on the LDL-R pathway, is associated with scarce effects on LDL-C reduction. The therapeutic strategies in this group of patients do not act on the LDL-receptor [72].

## 5. PCSK9 Monoclonal Antibodies’ Indications for Cardiovascular Secondary Prevention in Chronic Setting

PCSK9 monoclonal antibodies successfully showed, in cardiovascular outcome trials, to consistently reduce, in the context of secondary prevention, the risk for ASCVD. The efficacy of soluble PCSK9 inhibition for secondary ASCVD prevention is supported by two large cardiovascular outcomes trials—the ODYSSEY OUTCOMES and the FOURIER [41,42].

The efficacy and safety of alirocumab was evaluated in the ODISSEY OUTCOMES randomized placebo-controlled trial, which included 18,924 post-ACS patients. Over a 2.8 year follow-up, the primary outcome (composite of death for coronary heart disease, non-fatal MI, fatal or non-fatal ischemic stroke, or unstable angina requiring hospitalization) for alirocumab vs. placebo was 9.5% vs. 11.1%, respectively, resulting in a relative risk reduction of 15% (absolute risk reduction 1.6%) [42].

Among secondary outcomes, the alirocumab group showed lower major coronary heart disease and cardiovascular events but not mortality reduction.

Likewise, in the FOURIER trial, 27,564 patients with clinical ASCVD and LDL-C ≥ 70 mg/dL or a non-HDL-C level ≥ 100 mg/dL on mostly high intensity statin ± ezetimibe were randomized to evolocumab 140 mg subcutaneously every 2 weeks or 420 mg monthly versus placebo. At a median follow-up of 2.2 years, there was a 15% relative risk reduction (1.5% absolute risk reduction) in the primary composite outcome of cardiovascular death, myocardial infarction, stroke, hospitalization for unstable angina, or coronary revascularization in the evolocumab group compared with controls. Among individual endpoints, those randomized to evolocumab had significantly lower rates of myocardial infarction (MI), strokes and coronary revascularization but did not have significant difference in cardiovascular deaths compared with placebo. More recently, the results from FOURIER-OLE (open label extension) were presented at the ESC 2022 congress. FOURIER-OLE was therefore conducted to better understand the long-term safety, tolerability, and risk of major cardiovascular events in patients receiving prolonged treatment with evolocumab. The median follow-up in the extension study was 5.0 years. The maximum exposure to evolocumab in FOURIER plus FOURIER-OLE was 8.4 years. Long-term LDL cholesterol lowering with evolocumab was safe and well tolerated for more than eight years and led to further reductions in cardiovascular events compared with delayed treatment initiation [73]. Furthermore, evaluation of treatment with evolocumab on coronary artery plaque using intravascular ultrasound among statin-treated individuals, in the GLAGOV randomized controlled trial, found a modest but significant reduction in percent atheroma volume (−1.0%, 95% CI: −1.8% to −0.64%). After 76 weeks of treatment, there was also a greater proportion of patients exhibiting plaque regression in the evolocumab group compared with control (64.3% vs. 47.3%) [59].

## 6. Very Early Treatment of Acute Coronary Syndrome with Monoclonal Antibodies of Soluble PCSK9

Beyond the improvement in percutaneous revascularization techniques and antithrombotic strategies that contributed to change the scenario of acute coronary syndromes (ACS) [74,75,76,77,78,79,80,81], large attention has been paid to immediate and aggressive lipid-lowering therapies with high-intensity statins that are strongly recommended after ACS by the ESC/EAS dyslipidemia guidelines. Because of the delayed onset of action of statins and the risk of event recurrence during the first weeks after ACS, rapid lowering of LDL-C below the recommended targets might be beneficial in this group of patients. However, whether non-statin lipid-lowering agents could induce a further reduction in the risk of major adverse cardiovascular events is still unknown [82].

The use of anti-PCSK9 therapies, in the currently recommended guidelines, has not been included in the short-term after ACS but only after 4–12 weeks if the LDL-C target is not reached with oral therapies [69].

Moreover, the two main trials that investigated the anti-PCSK9, ODYSSEY OUTCOMES and FOURIER trials did not include patients with recent ACS (within 30 days of randomization) [41,42,83].

Nevertheless, recent trials have assessed the use of evolocumab and alirocumab early after an ACS.

The EVOPACS (Evolocumab for Early Reduction of LDL Cholesterol Levels in Patients with Acute Coronary Syndromes) trial evaluated the feasibility, safety, and LDL-C-lowering efficacy of in-hospital administration of evolocumab (<72 h from ACS symptom onset). Eligible patients were randomly assigned through a 1:1 ratio to receive evolocumab 420 mg every 4 weeks or matching placebo, on top of atorvastatin 40 mg. The reduction in LDL-C levels was significantly evident at 4 weeks and was maintained at 8 weeks. LDL-C was reduced below target (<55 mg/dL) in 90.1% of patients in the evolocumab group as compared with 10.7% in the placebo group, with no differences in terms of safety between the two groups [84].

In addition, the EVACS (Evolocumab in Acute Coronary Syndrome) trial enrolled patients with NSTEMI within 24 h of presentation. In-hospital initiation of evolocumab 420 mg rapidly reduced LDL-C, and at day 3, LDL-C levels in the group treated with evolocumab were significantly lower than those in the placebo group (49.2 +/− 24 vs. 76.1 +/− 33 mg/dL), remaining lower until day 30 (Table 1) [67].

These two studies were not powered for cardiovascular outcomes or serious adverse events, and thus, this point needs further investigation.

Based on this consideration, the EMSIACS (Evolocumab added to Moderate-Intensity Statin Therapy on LDL-C lowering and Cardiovascular Adverse Events in Patients with ACS) trial is an ongoing trial evaluating the efficacy of evolocumab added to moderate-intensity statin therapy early after ACS in adult Chinese patients, on both LDL-C lowering effect and cardiovascular outcomes after 12 weeks and 1 year of treatment [68].

The risk of recurrent events, in patients who experienced ACS, is largely attributable to frequent coexistence of multiple nonobstructive lesions in non-culprit arteries.

The PACMAN-AMI (Effects of the PCSK9 Antibody Alirocumab on Coronary Atherosclerosis in Patients with Acute Myocardial Infarction) trial and the HUYGENS (High-Resolution Assessment of Coronary Plaques in a Global Evolocumab Randomized Study) trial evaluated the effect of early administration of alirocumab and evolocumab, respectively, on coronary atherosclerosis. It was assessed by intracoronary imaging (OCT for both trials, IVUS and NIRS only for PACMAN-AMI) of the non-culprit arteries in patients presenting with ACS, after a year of treatment in addition to high-intensity statin therapy. These studies demonstrated a trend toward plaque stabilization, with significant reduction in percent of atheroma volume (assessed by IVUS), reduction in maximum lipid core burden index within 4 mm (assessed by NIRS), and increase in minimal fibrous cap thickness (assessed by OCT) [56,85], confirming, also in the acute setting, the results of the GLAGOV trial [59].

None of these trials investigated cardiovascular outcomes, but it is likely that LDL-C lowering and plaque regression will improve clinical outcomes in this population.

## 7. Prescriptive Flow-Chart and Real-World Adoption

“The lower the better” has been the conceptual goal that led to the publication of the latest ESC/EAS dyslipidemia guidelines in 2019. It emphasizes the proportional reduction of CV events associated with absolute reduction of LDL-C. According to this, a more important role has been attributed to PCSK9 monoclonal antibodies in primary and secondary prevention [66].

In patients who do not achieve therapeutic LDL-C goal, first-line therapy remains maximally tolerated statins, with or without ezetimibe. However, data from different registries show that this association alone is not enough to reach the goal, and PCSK9-i would guarantee a huge benefit for this population. Consequentially, PCSK9i prescription has been introduced, with different classes of recommendation, in different clinical settings [66].

In high-risk CV patients who do not reach their LDL-C goal with high-intensity statin and ezetimibe, the use of PCSK9-i is recommended in secondary prevention (I, A), while it could be useful in primary prevention (IIb, C) [66].

New attention has been paid to the setting of the ACS and to the early use of PCSK9i. ODYSSEY OUTCOMES and FOURIER showed that in this setting of patients, PCSK9i therapy is associated with a significant reduction of absolute risk of ischemic events and overall mortality [41,42].

**Figure 2:**
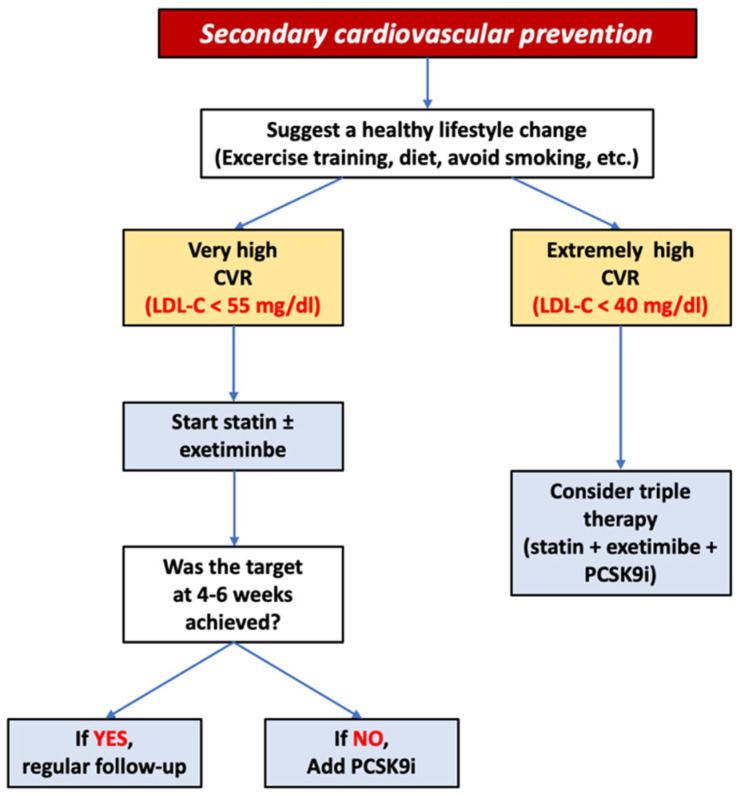
Flowchart of interventions for secondary cardiovascular prevention according to the European Society of Cardiology guidelines. CVR: cardiovascular risk, LDL-C: LDL cholesterol.

Therefore, in high-risk patients who have experienced an ACS, not reaching their therapeutic goal, the ESC/EAS guidelines recommend high-intensity statin and a new evaluation of LDL-C serum levels after 4–6 weeks. If LDL-C goal is not achieved, PCSK9i could be added as a combination therapy [66] (Figure 2).

Nevertheless, this gap in time might allow for a new CV event to occur with an avoidable increase in CV mortality, as several studies have shown [86].

Therefore, does a stepwise approach still make sense in this population? This is the reason why new guidelines suggest, for patients who present with an ACS and whose LDL-C levels are not within ideal parameters, despite already being on maximally tolerated statin dose and ezetimibe, the addition of a PCSK9 monoclonal antibody soon after the event (better during hospitalization if possible) (IIa, C).

Finally, FH, PAD and patients with statin-associated muscle symptoms (SAMS) are other high-CV-risk categories for which PCSK9i should be considered in association with high-intensity statin (without in case of SAMS) and ezetimibe to lower the LDL-C serum levels of patients who do not reach their goal [62,87,88,89].

Despite this and well-known higher therapeutic adherence, real-world data suggest that the use of PCSK9 therapy in daily practice is still very limited, probably for different reasons [90].

The first limit is that only few “prescription hubs” are available, despite the large number of eligible patients. Moreover, there are few local pharmacies where it is possible to withdraw these drugs. Finally, only a small register through which monitoring drug prescription, tolerability and safety is currently fillable. All these barriers dampen the extensive use of these drugs, as the DA VINCI study has shown. Even if the use of this therapy could have a significant positive impact on global cardiovascular health and on the economy of national healthcare systems, there is still need for great effort to ensure the outreach of these drugs to eligible patients [91].

## 8. Future Directions

Use of PCSK9-i helps to rapidly reach very low values of LDL-C, reducing the global CV risk, and confirming the postulate that not only “the lower”, but also “the earlier” is of crucial importance.

In the near future, it is likely that patients at very high risk with not-a-target LDL-C values will start directly with triple association (statin plus ezetimibe plus PCSK9-i) [91].

In the acute setting, it is presumable that the next set of guidelines will allow for the administration of PCSK9-i in the catheter lab during PCI, introducing the temporal concept of “door to PCSK9-i”, similar to the already known “door to balloon” concept.

The objective of reducing PCSK9 action to control LDL-C levels can also be achieved by inhibiting hepatic PCSK9 synthesis via targeted approaches to transcriptional regulation. The employment of small interfering ribonucleic acid (siRNA) oligonucleotides as pharmacological agents is appearing on the modern therapeutic scene.

Inclisiran is a double-stranded, chemically synthesized, long-acting anti-PCSK9 siRNA molecule. The antisense strand corresponds to PCSK9 mRNA, while the sense strand is conjugated with triantennary N-acetylgalactosamine carbohydrates (tri-GalNAc). This one is recognized by hepatocyte-specific asialoglycoprotein receptors. In hepatocytes, inclisiran activates the RNA-induced silencing complex (RISC), which promotes cleavage of the intracellular PCSK9 mRNA, inhibiting the protein translation of PCSK9 itself [92].

There are significant differences between the two approaches of PCSK9 inhibition that might eventually prove to have clinical relevance. Antisense intervention (a) mimics true relative PCSK9 deficiency seen in patients with PCSK9 LOF, (b) reduces intracellular levels of PCSK9, (c) shows no blockade of PCSK9 in the atheroma, (d) and does not induce nonphysiologic immune complex circulation. Entry of the siRNA approach (inclisiran) for PCSK9 inhibition into the marketplace could change the ecosystem of cholesterol therapies even more than the PCSK9-inhibiting antibodies have. This injectable agent could not only exhibit an even higher profile of efficacy and safety, but it is approved as a two-injection per year intervention, showing excellent adherence to the treatment [93].

Other approaches include oral small-molecule inhibitors, vaccination with the development of natural antibodies against PCSK9 and genic therapy with editing of the PCSK9 gene using clustered regularly interspaced short palindromic repeats (CRISPR) [94].

Vaccines. Two PCSK-9 vaccines (AT04A and AT06A) have been developed. They elicit the production of natural antibodies against PCSK9. Further investigations are needed for the durability of their effects [95].

CRISPR/Cas system (clustered regularly interspaced short palindromic repeats). This is a gene-editing technology that aims to introduce permanent genomic changes to alter gene function. The complex CRISPR/Cas introduces a double-strand break in the PCSK9 gene. Endogenous DNA repair mechanisms lead to random insertions or deletions, which render the gene dysfunctional [96].

Monobodies. Adnectin BMS-962476 is a small protein that inhibits free extracellular PCSK9. The drug is in preclinical phase but has shown, in non-human primates, adequate efficacy in reducing LDL [97].

Preclinical PCSK9 Inhibitors. EGF-A are mimetic peptides that block free PCSK9, enhancing, in vitro, LDL-R expression on the cell surface [98].

Several clues suggest that PCSK9 represents a pivotal factor in cardiovascular disease, in part independent from its effects on lipid metabolism. Statins, for example, have many pleiotropic effects on endothelial function, immune responses, inflammation, and thrombosis [99]. PCSK9-I, with a reasonable probability, has similar additive effects [100]. Results from trials are not univocal. Further research is needed to unveil pleiotropic activities of these drugs and to extent indications to other clinical conditions.

Today, the role of PCSK9 inhibition in lowering cardiovascular risk is well delineated. The modes of inhibition are increasingly being refined as well as the indications, which are being expanded and tailored. Future strategies for lowering CLDL and cardiovascular risk will allow for more scenarios to be attacked safely and effectively.

## Figures and Tables

**Table 1:** List of trials on PCSK9i with characteristics of patients’ population, number of patients enrolled and percentage of LDL-C reduction achieved. ACS: acute coronary syndrome; CV: cardiovascular; HeFH: heterozygous familiar hypercholesterolemia; HoFH: homozygous familial hypercholesterolemia; IRA: infarct related artery; LLT: lipid lowering therapy; NK: not known; Q2W: once every 2 weeks; SC: subcutaneous.

Trial Name	Patients’ Population	Patients	Treatments	LDL Reduction
**ALIROCUMAB**				
*ODYSSEY OUTCOMES* [46]	Prior ACS (1–12 months earlier) and LDL > 70 mg/dL on statin therapy	18924	Alirocumab 75 mg Q2W vs. placebo	55%
*ODYSSEY MONO* [47]	Hypercholesterolemia (with or without statin)	103	Alirocumab 75/150 mg and oral placebo vs. Ezetimibe and subcutaneous placebo	47.2%
*ODYSSEY ALTERNATIVE* [44]	Moderate (or higher) CV risk with statin intolerance	251	Alirocumab 75/150 mg plus oral placebo vs. ezetimibe plus SC placebo vs. atorvastatin 20 mg plus SC placebo	52.2%
*ODYSSEY COMBO I* [48]	High CV risk in uncontrolled hypercholesterolemia on maximally tolerated statin therapy +/− other LLT	316	Alirocumab 75/150 mg Q2W vs. placebo	48.2%
*ODYSSEY COMBO II* [49]	High CV risk in uncontrolled hypercholesterolemia on maximally tolerated statin therapy +/− other LLT	720	Alirocumab 75 mg Q2W (plus oral placebo) vs. ezetimibe (plus SC placebo)	50.6%
*ODYSSEY OPTIONS I* [50]	Very high CV risk and LDL > 70 mg/dl or high CV risk and LDL > 100 mg/dl on atorvastatin 20/40 mg	355	Alirocumab 75 mg Q2W vs. ezetimibe vs. double-dose atorvastatin vs. switch to rosuvastatin 40 mg	44.1/54%
*ODYSSEY OPTIONS II* [51]	Very high CV risk and LDL > 70 mg/dl or high risk and LDL > 100 mg/dL on rosuvastatin 10/20 mg	305	Alirocumab 75 mg Q2W vs. ezetimibe vs. double-dose rosuvastatin	50.6/36.3%
*ODYSSEY HIGH FH* [52]	HeFH and LDL > 160 mg/dL on maximally tolerated statin +/− other LLT	107	Alirocumab 150 mg Q2W vs. placebo	46%
*ODYSSEY LONG TERM* [53]	High CV risk and LDL > 70 mg/dL on maximally tolerated statin +/− other LLT	2341	Alirocumab 150 mg Q2W vs. placebo	62%
*ODYSSEY FH I and II* [54]	HeFH and uncontrolled LDL on maximally tolerated statin +/− other LLT	486/249	Alirocumab 75/150 mg Q2W vs. placebo	57.9/51.4%
*ODYSSEY CHOICE II* [55]	Statin intolerance and hypercholesterolemia	233	Alirocumab 150 mg Q4W or 75 mg Q2W vs. placebo	51.7/53.5%
*PACMAN-AMI* [56]	Intracoronary imaging of non-IRAs in early ACS on rosuvastatin 20 mg	300	Alirocumab 150 mg Q2W vs. placebo	−54.7 mg/dL
**EVOLOCUMAB**				
*FOURIER* [57]	Prior CVD and LDL > 70 mg/dL Background therapy of statin +/− ezetimibe	27564	Evolocumab 140 mg Q2W or 420 mg monthly vs. placebo	59%
*DESCARTES* [58]	LDL > 75 mg/dL Background therapy of statin +/− ezetimibe	901	Evolocumab 420 mg monthly vs. placebo	55%
*GLAGOV* [59]	Angiographic coronary disease and LDL > 80 mg/dL or 60-80 mg/dL plus any risk factor	968	Evolocumab 420 mg monthly vs. placebo	−56.5 mg/dL
*GAUSS-2* [60]	Hypercholesterolemia with statin intolerance	307	1. Evolocumab 140 mg Q2W or 420 mg monthly2. Ezetimibe3. Oral and subcutaneous placebo	54%
*MENDEL-2* [61]	Hypercholesterolemia (LDL > 100 mg/dL) and CV risk < 10%	614	1. Evolocumab 140 mg Q2W or 420 mg monthly2. Ezetimibe3. Oral and subcutaneous placebo	58%
*RUTHERFORD-2* [62]	HeFH and LDL > 100% Background therapy of statin +/− ezetimibe	331	Evolocumab 140 mg Q2W or 420 mg monthly vs. placebo	56%
*LAPLACE-2* [63]	Hypercholesterolemia Background therapy of statin +/− ezetimibe	2067	1. Moderate-intensity vs. high-intensity statin2. Evolocumab 140 mg Q2W or 420 mg monthly3. Ezetimibe4. Oral and subcutaneous placebo	70%
*TESLA Part B* [64]	HoFH and LDL > 130 mg/dL on maximal therapy	50	Evolocumab 420 mg monthly vs. placebo	23,1%
*OSLER-1 and OSLER-2 (open label)* [65]	Hypercolesterolemia on standard therapy	4465	Evolocumab 420 mg monthly or 140 mg Q2W plus standard therapy vs. standard therapy alone	61%
*HUYGENS* [66]	Intracoronary imaging of non-IRAs in early ACS on maximally tolerated statin therapy	150	Evolocumab 420 mg monthly vs. placebo	NK
*EVOPACS* [67]	In-hospital phase of ACS on atorvastatin 40 mg	308	Evolocumab 420 mg monthly vs. placebo	40,7%
*EVACS* [68]	In-hospital phase of ACS on maximally tolerated statin therapy	60	Evolocumab 420 mg vs. placebo	−27 mg/dL

## Data Availability

Not applicable.
